# Airborne Biogenic Particles in the Snow of the Cities of the Russian Far East as Potential Allergic Compounds

**DOI:** 10.1155/2014/141378

**Published:** 2014-07-22

**Authors:** Kirill S. Golokhvast

**Affiliations:** ^1^Scientific Educational Center of Nanotechnology, Far Eastern Federal University, 10 Pushkinskaya Street, Vladivostok 690950, Russia; ^2^Scientific Research Institute of Medical Climatology and Rehabilitation, Vladivostok Branch of Far Eastern Scientific Center Physiology and Pathology of Breathe, 73 Russkaya Street, Vladivostok 690105, Russia

## Abstract

This paper presents an analysis of airborne biogenic particles (1 mkm–1 mm) found in the snow in several cities of the Russian Far East during 2010–2013. The most common was vegetational terraneous detritus (fragments of tree and grass leaves) followed by animal hair, small insects and their fragments, microorganisms of aeroplankton, and equivocal biological garbage. Specific components were found in samples from locations close to bodies of water such as fragments of algae and mollusc shells and, marine invertebrates (needles of sea urchins and shell debris of arthropods). In most locations across the Far East (Vladivostok, Khabarovsk, Blagoveshchensk, and Ussuriysk), the content of biogenic particles collected in the winter did not exceed 10% of the total particulate matter, with the exception of Birobidzhan and the nature reserve Bastak, where it made up to 20%. Most of all biogenic compounds should be allergic: hair, fragments of tree and grass leaves, insects, and microorganisms.

## 1. Introduction

Suspended particulate matter in air as abiotic factor has not been studied until recently. Its formation is a complex and multifaceted process that generally depends on soil erosion, volcanic activity, fire, and global air-mass circulation. In addition to quantifying air pollution (commonly measured by weight), which is occasionally the only criterion, particle size and composition in suspended material are critical for air monitoring.

For a long time, since the planetesimal occurrence that eventually became the Earth, air suspensions included predominantly materials other than organic (mineral and rock particles) and low-molecular organic compounds drawn by meteorites and asteroids. The recently discovered planetesimal (e.g., Lutecia 21) is now covered with nonorganic dust (layers up to 600 m) [1, 2], which arrived at the start of the solar system evolution.

Over the course of time, or more precisely, since the appearance of life on Earth and accumulation of large biomaterial deposits, air suspensions became enriched with residues of complex polymer organic matter, which was also constantly changing, being a dynamic system.

It should be noted that the particle composition in the snow samples is different from the samples collected in spring and summer which had an abundance of plant pollen in them [3]. Pollen in particular, as many researchers suggest, is believed to cause allergic diseases, whose frequency typically peaks from the middle of May to the middle of August. That is why the majority of research work to date deals with highly allergenic compounds in air suspensions (or aeroallergens) like fungi spores and plant pollen, produced during warm seasons [3–12].

There are also reports about other aeroallergens—hair of cats and dogs and dust mites [13–21].

We have already discussed the issue of differences between natural and industrial suspensions across the Far East region and the ecological and hygienic value of this [22].

This paper considers a material research of biogenous particles in air suspensions found in snow samples from the urban areas in the Russian Far East to provide information about potential allergic features.

## 2. Materials and Methods

The methodology used to study airborne biogenous particles is commonly centered on analyzing plant pollen [23, 24], whereas other components need different techniques to be applied.

We have studied the airborne suspensions that were found in snow samples collected during 2010–2013 in the biggest cities in the Russian Far East: Vladivostok (at 13 points), Khabarovsk (12 points), Birobidzhan (5 points), Blagoveshchensk (25 points), Ussuriysk (10 points), and National Reserve Bastak (5 points).

To avoid secondary pollution, we collected the samples (atmospheric precipitation of ice crystals) only in snowfalls. Only the upper layer (5–10 cm) of new snow was used for this purpose. The snow was then placed in three-litre sterile containers. The containers were cleaned of dust with distilled water before use. The liquid received from melted snow was dried out in sterile and dust-free conditions. The light microscope Nikon SMZ1000 and the electronic microscope Hitachi S-3400N, equipped with an energy-dispersive spectrometer (EDAX, Thermo Scientific), were used to analyse airborne suspensions taken from those containers. Fifty particles were selected for the field of view for every sample. Biogenous particles were then singled out based on the morphology and the results of energy-dispersive analysis. Platinum was used to sputter coating and examine the samples under the electronic microscope.

## 3. Results

The biogenous particles found in the analyzed samples were classified as follows: vegetational terraneous detritus (fragments of tree and grass leaves), animal hair, small insects (lice, fleas) and their fragments, aeroplankton, and equivocal biological garbage.

**Figure 1:**
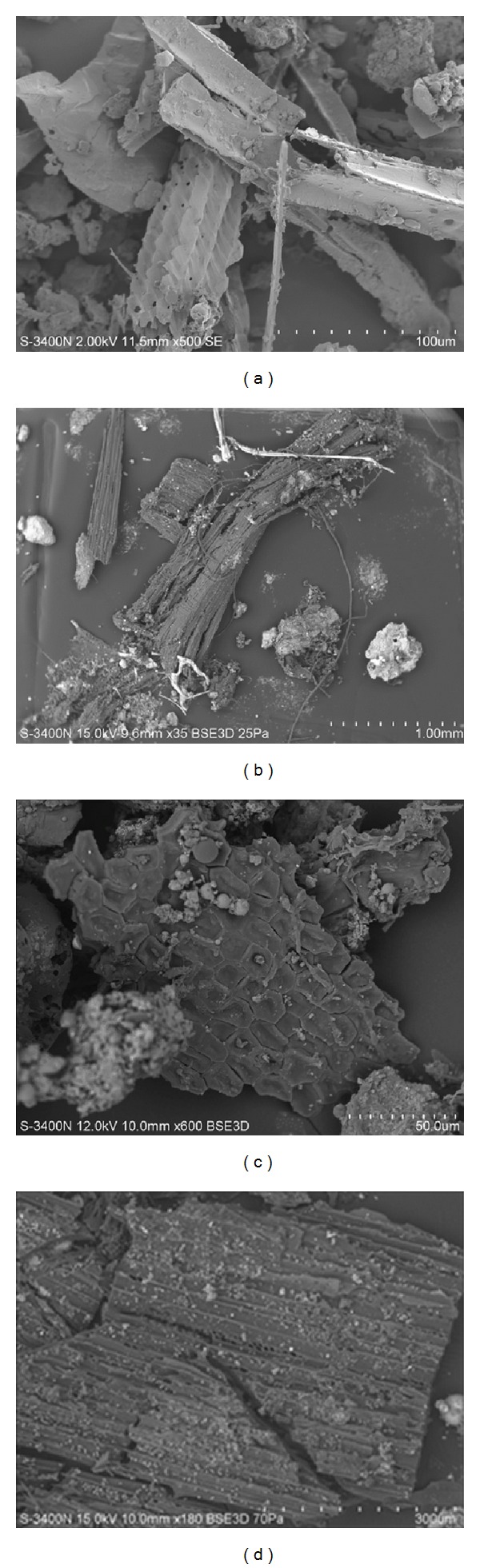
Electronic micrography: (a) fragments of leaves from a snow sample taken in the area of the ring motorway in Birobidzhan. Magnification ×500. (b) Wood particles from a snow sample taken at the exhibition area in Blagoveshchensk. Magnification ×35. (c) Fragments of leaves from a snow sample taken in the center of Bastak nature reserve. Magnification ×600. (d) Wood particles from a snow sample taken at the intersection of Partizanskaya and Lenin streets in Blagoveshchensk. Magnification ×180.

Vegetational terraneous detritus was the most frequent, even in winter, when its amount was up to 70%. Particles of vegetational terraneous detritus mostly present themselves as undefinable materials of leaves, stems (Figures 1(a), 1(c), and 1(d)), wood (Figure 1(b)), diatoms, and phytolithic shells.

Phytoliths (biominerals of plants) and shell debris of diatoms were the rarest particle types in the samples. The snow samples occasionally included plant pollen, whose content was several times higher in the spring and summer periods.

### 3.1. Animal Hair

**Figure 2:**
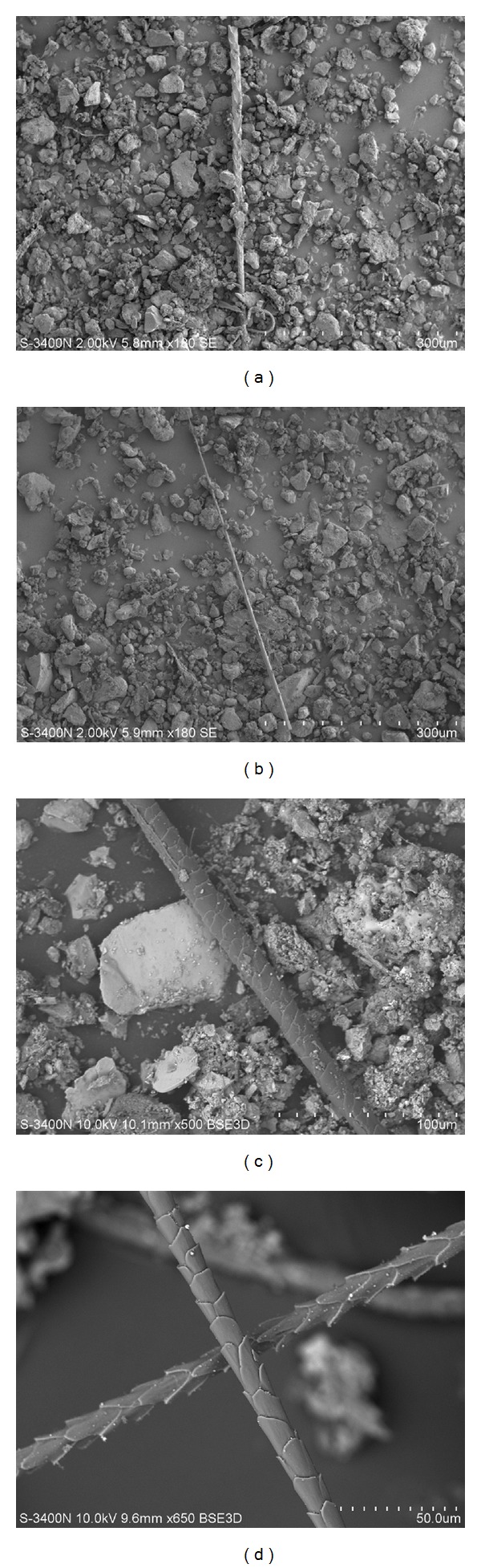
Scanning electron microscopy in secondary electrons: (a) and (b)—hair of unknown animal (sheep* Ovis aries*?) from a snow sample collected in Zmeinka District, Vladivostok—; the sample was taken during a dust storm in Mongolia 04.09.2012. Magnification ×180. (c) Hair of a dog (*Canis familiaris*) from a snow sample taken in a one district of Blagoveshchensk. Magnification ×500. (d) Hair of a cat (*Felis domesticus*) from a snow sample taken around the Main Railway Station, Blagoveshchensk. Magnification ×650.

Hair of animals has a pronounced human health effect (in the form of allergies) as a factor in the presence of domestic animals (pets) at home [25]. Figures 2(a)–2(d) show that this type of airborne allergen can also be found outdoors.

Hairs of animal species were identifyed using [26, 27].

The allergic response to pets in humans, that is, to hair of pets, is known to be one of the most challenging issues in the modern allergy science [14, 15, 17–20]. Allergens of cats are the best studied types [21].

Gusareva and coauthors, for example, [17] note that most cases of bronchial allergy in patients (57.3%) have sensitization to the primary allergen of cats (Fel d 1).

Diette and coauthors [25], in their study, provide a sensitization correlation diagram for the Fel d 1. It should be noted that the circle of allergens relative to hair of animals has become wider more recently.

In addition to allergens in cats—Fel d 1, Fel d 2 (albumin), Fel d 4 (lipocalin), Fel d 5w [14, 16, 21]—, those in dogs have already been described, such as Can f 1 [13].

### 3.2. Small Insects

**Figure 3:**
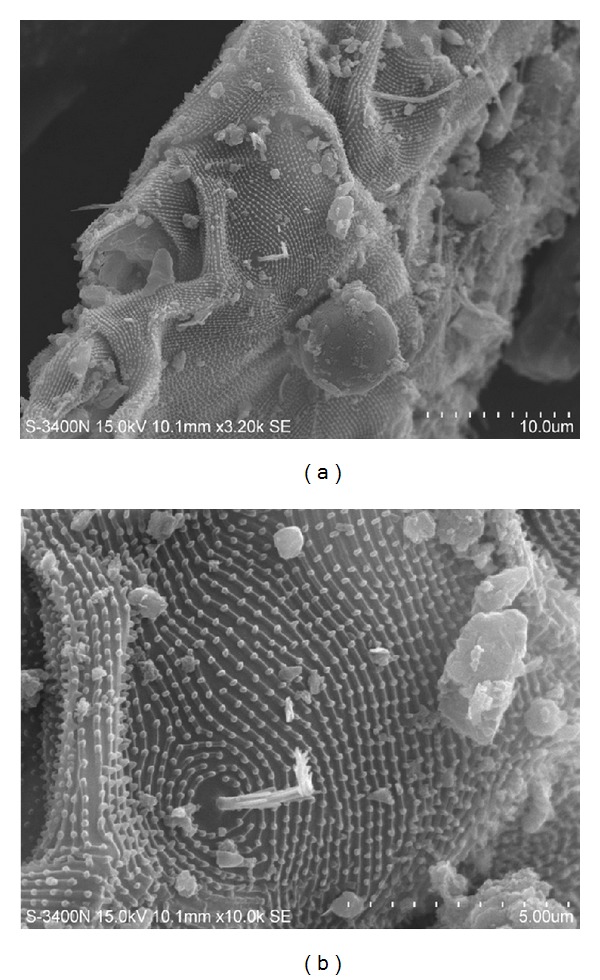
(a) Fragments of unknown insect from a snow sample taken in the area of the Ring Motorway in Birobidzhan. (a) Magnification ×3200. (b) Magnification ×10000.

Small insects in airborne suspensions are a rare event (Figure 3), but if we consider their higher allergenic potential, this type of particle deserves a closer look.

Except for allergens of dust mites (Der p 1, Der f 1), there are also descriptions of allergic reactions to allergens of other insect species, for example, of cockroaches (Bla g 2) [13, 28, 29].

According to data from Gusareva and coauthors [17], almost one-third of bronchial allergy cases has sensitization to allergens of dust mites, while Huss and coauthors [28] report on the extremely high level of mite contaminations in dust from households in a few cities of the United States and Canada. For example, in San Diego, this level is 78.5% and in Toronto 59.3%. According to further investigation by this author, the presence of cockroach allergen in households of Boston was registered in 21.5% of the study cases, Saint Louis 16.3%, and Baltimore 13.4%.

Taking into account this fact, we can suggest that the air in these cities will be abundant with such allergens too.

### 3.3. Aeroplankton

Particles of air suspensions often become a living environment for a wide range of organisms—bacteria and fungi—some of which are sources of toxins, dangerous to human health [6, 30]. In general, all airborne living organisms (bacteria, fungi, moss, algae, spores, pollen, phytoplankton, tiny seeds, and arthropods) can be considered as aeroplankton [31]. Spores of many bacteria easily reach high atmospheric strata and can spread over large areas [32]. Aeroplankton in combination with dust is believed to seriously influence the weather; it often becomes a centre for atmospheric ice desublimation in particular [33]. Klyuzko and coauthors [34] demonstrated that, under laboratory conditions, bacteria contribute to the freezing of water by acting as typical condensation nuclei. Aeroplankton are found even at a height of 9000 meters [35].

**Figure 4:**
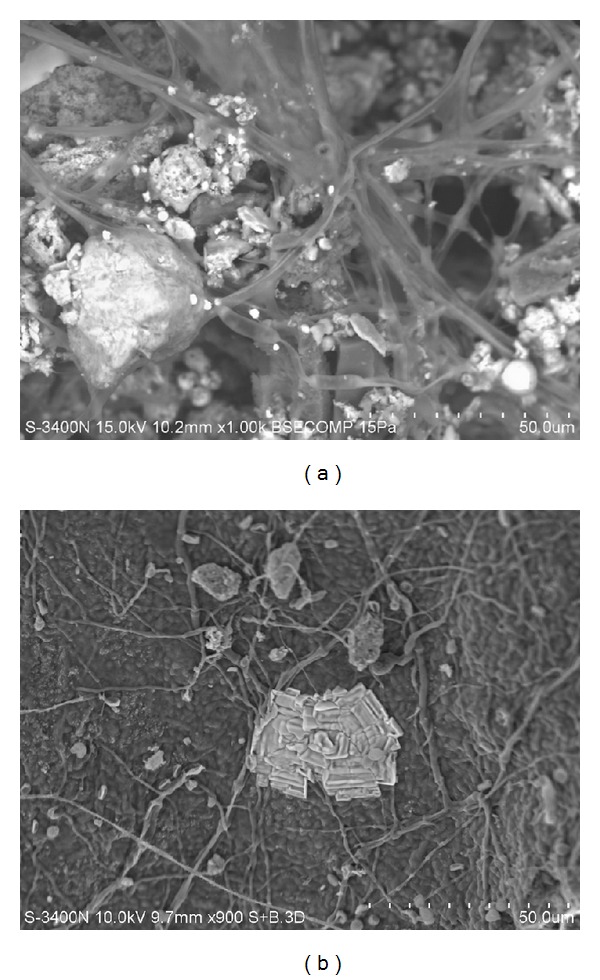
Hyphae of fungi from a snow sample taken: (a) at the Pervomayskiy park area in Blagoveshchensk, (b) at the Child Sanatorium “Detskiy” in Khabarovsk. Scanning electron microscopy. (a) Magnification ×1000. (b) Magnification ×900.

We found some hyphae of unknown fungi in the snow sample taken in the Pervomayskiy park area in Blagoveshchensk (Figure 4(a)) and in Child Sanatorium “Detskiy” in Khabarovsk (Figure 4(b)).

The presence of water, micronutrients, oxygen, carbon oxide, and nitrogen inside clouds, as well as the presence of intensive radiation energy, creates favourable conditions for photosynthesis and metabolism and cell growth [36]. This is why the atmosphere and, more precisely, clouds represent a unique environmental system [37, 38], which influences the composition and properties of airborne solid particles.

Due to this fact, aeroplankton can be regarded not only as a carrier of various allergens but also as a potential ecosystem for pathogenic germs.

### 3.4. Sea Detritus

**Figure 5:**
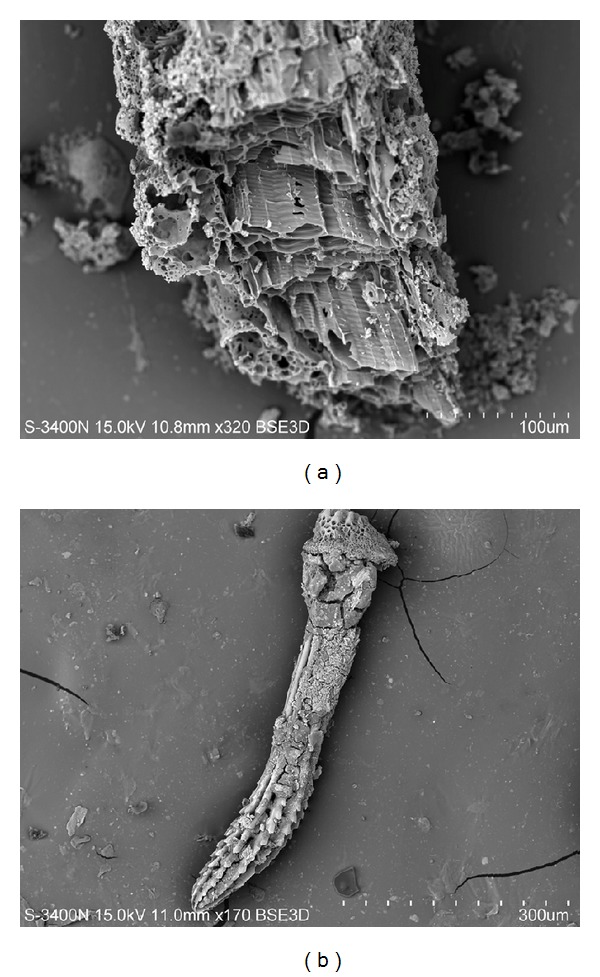
(a) Fragments of sea organics (hypothetically a part of a sponge) and (b) needle of sea urchin Scaphechinus mirabilis from a snow sample taken in the Sadgorod District of Vladivostok. Scanning electron microscopy in secondary electrons. (a) Magnification ×320. (b) Magnification ×170.

Any components of sea flora and fauna can be present in the air of seaside cities and settlements, especially if located close to the fish and seafood processing industry (Figures 5(a) and 5(b)).

Allergic reactions are most likely (and also are most studied) to the proteins of shrimps— tropomyosin (Pen a 1) [39], of fishes—, the cod allergen Cad c 1 [40, 41], and the perch allergen [42].

### 3.5. Equivocal Biological Garbage

**Figure 6:**
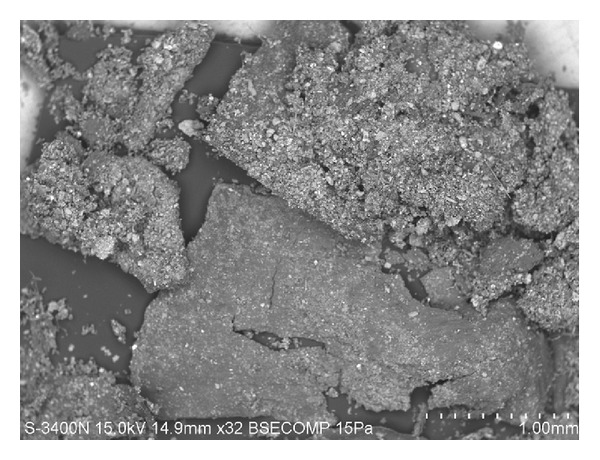
Aggregates from naturally occurring minerals, vegetational detritus, and equivocal biological garbage in a snow sample taken at the Pervomayskiy park area in Blagoveshchensk. Scanning electron microscopy in secondary electrons. Magnification ×32.

According to the elementary analysis, this component of airborne suspensions contains a large amount of carbon (max. 90%), but it is not homeomorphous like charcoal and soot (Figure 6).

In equally possible chances, such indefinable organic garbage may contain digested residues of any organic detritus, including that of industrial origin (e.g., food industry or sawmill wastes). Possible source of garbage is the 2 big rivers: Amur and Zeya.

## 4. Discussion

The results above provide enough data for judgments and allow us to specify the components by the order of their occurrence in airborne suspensions from the snow samples taken in various locations across the Far East region of Russia: vegetational terraneous detritus (fragments of tree and grass leaves), animal hair, small insects and their fragments, microorganisms of aeroplankton (bacteria and fungi), and equivocal biological garbage [43].

Considering the literature data, the airborne biogenous particles found in our samples have the following order by allergenic potential: animal hair, fragments of insects, vegetational detritus, and pollen. The role of industrial garbage that contains biological components in allergies is completely unknown as there are no means to identify its precise composition.

Basic biogenous particles in air suspensions can be singled out and specified separately across every location of the Far East region according to this study. Particles of sea and land detritus, for example, prevail in Vladivostok, a major city and harbor on the Japan Sea. Airborne biogenous particles in air suspensions from Khabarovsk and Blagoveshchensk (both of which are located on the banks of the Amur River and the Zeya River) also in most cases belong to land detritus.

A similar trend, that is, predominance of land detritus particles, is found in Birobidzhan and the State Natural Reserve of Bastak. However, it should be noted that in all tested locations naturally occurring minerals and rocks with insignificant organic ingredients (5–10%) dominated in the whole picture. The samples taken in Birobidzhan and the Bastak reserve are noted for the highest levels of vegetational detritus among all tested populated locations in the Far East, with a total amount of 20–30% in the particles. This is likely due to weather conditions, geographic location, and vast woodlands.

Equivocal biological garbage is prevailing in the air suspensions taken form Ussuriysk (a land locked town, average by size, with much pollution from industry and transport).

The analysis found particles of a biological origin and their possible allergic effect (considering quantity of finds) is provided in Table 1.

The share of biological components differs between the cities (5 to 25%), but we consider that settlements, where in air there are only a lot of parts of plants, threat of allergies will not be high.

The degree of “infection” of suspensions particles by aeroplankton was approximately equal in all cities (1 of 20–25 particles), but in Blagoveshchensk and Khabarovsk—is higher (1 of 10–15 particles). As shown in [44] patients with bronchial asthma in Amur region (Blagoveshchensk), mycotic allergy is accompanied by the domestic (air) sensitization. It is also worth noting that the surface of biogenous particles often serves as an adsorbent for nanoscale particles of both natural and artificial origins.

We consider that existence of large water objects allows effectively clearing the atmosphere of polluting particles, including biogenous origin. But, on the other hand, water objects can be a source of allergens.

According to our results, “winter” air suspensions contain a considerable amount of allergenic organic matter that is consistent with the data of other studies [45]. In the course of the analysis, [46] revealed a progressive increase in the incidence of complaints of child population of Khabarovsk for urgent and emergency allergy care.

As a whole, we see that in the cities of Vladivostok and Khabarovsk growth of allergic diseases [44, 46] is noted. Unfortunately data on allergies of other studied cities in literature are absent; therefore, it is not possible today to draw a conclusion on correlation between types of biological particles and allergies.

## Figures and Tables

**Table 1:** Influence of different factors on structure of biological components of suspensions.

Cities	Water object	Forest area	Mail biological components	Share, %	Level of allergic threat
Vladivostok	Seaside	Small	Plant detritus, sea detritus	5	Average
Khabarovsk	One big river	Average	Plant detritus, aeroplankton	5	Average
Birobidzhan	—	Big	Plant detritus	20	Small
Blagoveshchensk	Two big rivers	Average	Equivocal biological Garbage, plant detritus, and aeroplankton	10	High
Ussuriysk	Three small rivers	Small	Equivocal biological garbage	10	High
Bastak	—	Big	Plant detritus	25	Small
